# Reflexión crítica sobre la toma de decisiones en procedimientos quirúrgicos de terceros molares

**DOI:** 10.21142/2523-2754-1203-2024-203

**Published:** 2024-09-17

**Authors:** Raúl Andrés Montes-García, Xiomara Zilena Serpa-Romero, Jorge Homero Wilches-Visbal

**Affiliations:** 1 Universidad del Magdalena. Santa Marta, Colombia. raulmontesag@unimagdalena.edu.co , xserpa@unimagdalena.edu.co, jwilches@unimagdalena.edu.co Universidad del Magdalena Universidad del Magdalena Santa Marta Colombia raulmontesag@unimagdalena.edu.co xserpa@unimagdalena.edu.co jwilches@unimagdalena.edu.co

Los estudios describen la extracción de terceros molares como un procedimiento que amerita experiencia clínica y práctica quirúrgica a fin de disminuir los riesgos de complicaciones intraoperatorias y posoperatorias, como dolor, trismus, sangrado, infección, edema, lesiones del nervio alveolar inferior, desplazamiento de los dientes a espacios vecinos y fractura mandibular en los casos más severos ^(1, 2)^. Después de revisar algunas de las consecuencias más comunes en la práctica odontológica, se observa que entre los aspectos relevantes del protocolo quirúrgico se incluyen la destreza clínica, la complejidad quirúrgica, el estudio radiográfico y el diagnóstico definitivo ^3^.

El código de ética del odontólogo en Colombia indica que “todo profesional de la odontología es responsable de los procedimientos que realice, los cuales deben contar con el consentimiento informado del paciente, actuar según sus competencias y responsabilizarse por las complicaciones, debido a que el desconocimiento de la ley no lo exime de responsabilidad” ^4^, razón por la cual se debe reflexionar acerca del nivel de competencias antes de realizar estos procedimientos. El odontólogo general que pretenda realizar una cirugía de terceros molares debe estar seguro de sus habilidades y limitaciones, además de considerar que, en los casos de mayor complejidad, se hace imprescindible remitir a especialistas como los cirujanos orales o maxilofaciales. 

En efecto, en una investigación realizada por Sánchez *et al*. ^1^, estos mencionan que la formación académica odontológica es un dato que influye de forma significativa en la dificultad percibida para la extracción de terceros molares. Además, resaltan la relación entre los factores tomados en cuenta para determinar el nivel de dificultad de una cirugía oral, y que los especialistas refieren que los factores más importantes están relacionados con el paciente y su comportamiento, mientras que los odontólogos generales perciben como más importantes los relativos al procedimiento quirúrgico. 

Asimismo, en un estudio realizado por Kiencało *et al*. ^2^, se plantea que las complicaciones intraoperatorias asociadas con la extracción de terceros molares pueden incluir el daño del nervio alveolar inferior, tanto durante la aplicación de la anestesia como durante la propia extracción. La fractura de una muela del juicio o el daño de los dientes adyacentes, así como la fractura o luxación mandibular, o la fractura de la tuberosidad maxilar, suelen estar asociadas al uso de una fuerza excesiva. 

En un estudio retrospectivo realizado por Sayed *et al*. ^3^ se encontró que la tasa general de complicaciones posoperatorias fue del 8,3%. La extracción de terceros molares, a menudo, se asoció con dolor, hinchazón y *trismus* posoperatorios esperados y típicamente transitorios; sin embargo, en ocasiones, este dolor puede presentarse más allá de la primera semana posoperatoria y requerir tratamiento adicional, como la colocación de un vendaje o la administración de antibióticos durante una visita de seguimiento ^5^. En los casos que se revisaron en este estudio, los síntomas se resolvieron gradualmente con medidas de apoyo.

Es relevante establecer el nivel de dificultad quirúrgica en el preoperatorio, dado que es un paso esencial para garantizar una correcta planificación del tratamiento. Para ello, se recomienda emplear el índice de dificultad de Pederson, dado que esto permitirá a los clínicos preparar el equipo y los materiales adecuados, decidir el punto correcto de acceso quirúrgico, la técnica correcta, el tipo de anestesia, y determinar si las capacidades individuales del clínico están o no a la altura de la extracción en cuestión. Estas medidas conducirán a una intervención de menor riesgo y con menos complicaciones intra y posoperatorias ^1^. 

A partir de la evidencia presentada, se reflexiona sobre la importancia que el odontólogo general debe dar a los procedimientos de terceros molares incluidos y así realizar un análisis preparatorio teniendo en cuenta los siguientes aspectos:

1. Evaluar los casos de forma adecuada realizando una planificación, de acuerdo con la clasificación de Winter, Nolla, Pell y Gregory.
2. Establecer las enfermedades sistémicas presentes, la relación radiográfica con estructuras vecinas y el estado estructural del órgano dental por extraer, lo que permitirá puntualizar las competencias del profesional tratante.
3. Reconocer la importancia que tiene el marco ético y legal vigente, con el apoyo de los protocolos de manejo, sin excederse, y cumplir con la norma establecida.
4. Examinar las complicaciones que pueden presentarse durante el desarrollo del procedimiento. Por eso, es muy importante tener el conocimiento requerido para solucionar en el menor tiempo y con la técnica adecuada cualquier evento desfavorable o adverso, a fin de evitar secuelas en el paciente o posibles demandas por mala praxis o negligencia médica.
5. Incluir el uso de los antibióticos a manera de premedicación y medicación posoperatoria.
6. Evitar excederse en la realización de procedimientos que ameriten la intervención de un especialista y reconocer la necesidad de remisión a estos cuando no se tiene la habilidad clínica para abordar el problema.
7. Nunca olvidar que la práctica basada en la evidencia se establece como un elemento fundamental e indicador clave de una atención de alta calidad al paciente.

Esta es una secuencia de pasos sugerida que ayudará a tomar decisiones responsables y disminuir los riesgos y las posibles complicaciones de los procedimientos abordados por odontólogos generales en tratamientos quirúrgicos de terceros molares.
